# Social-Emotional Problems Among 3-Year-Olds Are Associated With an Unhealthy Lifestyle: A Population-Based Study

**DOI:** 10.3389/fpubh.2021.694832

**Published:** 2021-11-16

**Authors:** Eva Eurenius, Amal Farah Mohamed, Marie Lindkvist, Anneli Ivarsson, Inger Öhlund, Masoud Vaezghasemi

**Affiliations:** ^1^Department of Epidemiology and Global Health, Umeå University, Umeå, Sweden; ^2^Eating Disorder Unit, Helsinki University Hospital, Helsinki, Finland; ^3^Department of Clinical Science, Pediatrics, Umeå University, Umeå, Sweden

**Keywords:** ages and stages questionnaires, child behavior, cross-sectional studies, family characteristics, preschool children

## Abstract

**Introduction:** Little attention has been paid to the association between preschool children's social-emotional problems and lifestyle at the population level.

**Objective:** This study aimed to overcome this knowledge gap by investigating to what extent children's social-emotional problems are associated with their lifestyle and if there are any gender differences.

**Methods:** This cross-sectional, population-based study used data from the regional Salut Register in northern Sweden, including 7,179 3-year-olds during 2014–2017. Parents responded to a questionnaire including the 36-month interval of the Ages and Stages Questionnaires: Social-Emotional (ASQ:SE) and questions regarding family and lifestyle characteristics. Single and multiple logistic regression were used to assess the association between children's social-emotional problems and multiple family lifestyle characteristics.

**Results:** More reports of social-emotional problems were found among children who did not have parents living together or had markers of an unhealthy lifestyle. Children who ate vegetables less frequently, whose parent/-s brushed their teeth less often and did not read to them regularly were more likely to have social-emotional problems. Playing outdoors <3 h during weekdays and >1 h of sedentary screen time during weekends increased the risk of social-emotional problems among boys only, while >1 h of sedentary screen time during weekdays increased the risk among girls. When it comes to lifestyle and gender differences, a high proportion of the 3-year-olds had an unhealthy lifestyle, more so for boys than for girls. The dietary quality and tooth brushing were somewhat more adequate for the girls than for the boys, but boys spent more time playing outdoors compared to the girls.

**Conclusions:** This study provides us with an important overview picture of the family life situation of three-year-olds, including those with social-emotional problems. Such problems were significantly associated with markers of unhealthy lifestyle, with significant gender differences. Therefore, this study suggests that in order to maintain children's social-emotional ability and support children at risk of problems, public health intervention programs should have a broader perspective on improving children's lifestyle rather than merely focusing on their social and emotional problems, and the gender differences found may be taken in account.

## Introduction

The concept of social- and emotional ability includes the child's experience, expression, and management of emotions and the ability to establish positive and rewarding relationships with others (1). Childhood development underpins lifelong behavior, learning and health, and it has been strongly linked to mental health in adulthood (2–4). Preschool age is a usual age to find signs of social, emotional and behavior problems (4, 5). There are several epidemiological studies regarding psychosocial and behavioral problems among children within and between countries. Swedish children, as the other Scandinavian countries, have a low occurrence of parental-reported behavioral and emotional problems compared to many other countries (6). Almost one in ten Swedish 3-year-olds has social-emotional problems as reported by their parent/-s (7). Gender differences are evident with twice as many boys compared to girls having such problems, which has been reported earlier for this study group of children (boys 12.3 and girls 5.6%, *p* < 0.001) (7). During the last decade mental health complaints have increased among school-age children in Sweden (8) and mental health problems have become a public health concern. Therefore, it is imperative not only to detect children at risk as early as possible, but also to identify associated family and lifestyle characteristics.

A healthy lifestyle is a way of living that lowers the risk of developing non-communicable diseases, becoming seriously ill or dying early (9). When adopting a healthy lifestyle as an adult, a more positive role model is given to other members of the family, especially children (9). Unhealthy behavior or lifestyle during childhood has been shown to track into later life (10, 11). Healthy nutritional behavioral and physical active choices are vital in order for a child to grow into a healthy adulthood (12). Preschool-aged children who have sleeping problems (13, 14) or unhealthy feeding practices (15) experience social-emotional problems to a higher extent. Evidence on the role of physical activity and sedentary behavior for the mental health of preschool-aged children is nearly non-existent. However, results from observational studies suggest that promoting physical activity and decreasing sedentary behavior might protect mental health in children and adolescents (16). Some family characteristics are associated with social-emotional problems among children, e.g., exposure to domestic violence (17), depression among mothers (14, 18), and custody arrangements (7). Among many risk factors, early childhood caries is associated with family characteristics such as oral health and feeding behaviors (19).

Regardless of how lifestyle characteristics are causally related to social-emotional problems, it is pertinent to study the association between multiple lifestyle characteristics simultaneously and social-emotional problems, especially among preschool children at the population level where little is known. Neither unhealthy lifestyle behaviors, nor social-emotional problems among preschool children happen in isolation. The interrelation between these may reflect the broader family context in which children live and grow up and can help to inform public health policies aimed at improving children's health and wellbeing. Therefore, this study aimed to overcome this knowledge gap by addressing the associations between 3-year-olds' social-emotional problems and lifestyle taking into account any gender differences.

## Methods

### Study Design and Context

This population-based, cross-sectional study was performed in Västerbotten County in northern Sweden with an annual birth rate of about 3,000. It takes advantage of data from the Salut Child Health Promotion Program, an ongoing universal intervention and epidemiological survey of expectant parents and children (20). The data originates from a questionnaire used in 3-year-olds at the county's 40 Child Health Care centers. The parents fill in the questionnaire at home and brings it to the regular visit. The questionnaire is including the 36-month interval of the Ages and Stages Questionnaires: Social-Emotional (ASQ:SE) and questions regarding family and lifestyle characteristics. The questionnaire is used to increase nurses and parent's awareness of children's social and emotional problems and lifestyle, giving a possibility to identify children in need of extra support.

### Study Participants

From January 2014 to September 2017, 8,214 3-year-olds' parents responded to the questionnaire, corresponding to 80% of those living in the county. The age of each child was calculated by deducting the birth date from the Child Health Care centers' visit date. Only children within the questionnaires age range were included (33–41 months) (1). From the initial sample, 1,035 children were excluded due to any of these three reasons: the parent/-s did not consent to the research (*n* = 447); the age of the child could not be determined or was outside the recommended age range of the 36-month interval of the ASQ:SE (*n* = 513); and the number of unanswered ASQ:SE questions were more than three (*n* = 75). In total, 7,179 3-year-olds were included, corresponding to 70% of all children living in the county during the study period. A majority of the families lived in an urban area, and place of residence did not differ between boys and girls, which has been reported earlier (7).

### Data Collection Procedure

The first edition of the ASQ:SE 36-month interval was included in the questionnaire used in this study (1, 21). The ASQ:SE reflects the risk of social-emotional problems among children and it is considered to have adequate psychometric properties that includes validity and reliability internationally (7, 21–23). ASQ:SE consists of 31 items and three open-ended questions, the latter not used in this study. Out of the 31 ASQ:SE items, up to three missing values were replaced by using average value imputation from all the other responses, according to ASQ:SE User's Guide (1). A three-point Likert scale was used to enable parents to indicate how often they perceive their child's behavior, and whether the behavior is a concern for them. This results in a total score ranging 0–465 points. Children on the cut-off value of 59 points or above are considered to be at risk of social-emotional problems (1). A back-translation was used in order to get a Swedish version of the ASQ:SE, based on established recommendations (24). In addition to the ASQ:SE, the questionnaire included information on family characteristics such as the child's gender, custody arrangement, number of siblings, and attendance at preschool and different lifestyle characteristics. The child's parental-reported lifestyle (both at home and in preschool) do not go into details, but gives us an overview of the child's lifestyle. The lifestyle questions included dietary quality in terms of food-frequency for low-fat milk, vegetables, fruit/berries, fish and sweets/soft drinks. Physical activity and sedentary screen time were included in terms of frequency of time playing outdoors (weekdays and weekends) and sedentary screen time (during weekdays and weekends). The frequency of parents brushing the child's teeth and reading to the child was also parts of the lifestyle questions (Table 1).

**Table 1:** Characteristics of 3-year-olds within the Salut Child Health Programme (2014–2017).

**Characteristics**	**Boys, *n* (%) *n* = 3,719**	**Girls, *n* (%) *n* = 3,460**	***P*-value^**a**^**
Custody arrangement			0.091
Parents living together	3,327 (91)	3,147 (92)	
Parents not living together or single parent	315 (9)	257 (8)	
Having siblings			0.936
Yes	2,717 (75)	2,531 (75)	
No	887 (25)	830 (25)	
Attending preschool			**0.042**
Yes	3,587 (97)	3,368 (98)	
No	108 (3)	74 (2)	
Drinking low-fat milk			**0.009**
Twice a day or more often	2,444 (68)	2,342 (69)	
Once a day	581 (16)	582 (17)	
Less than once a day	584 (16)	461 (14)	
Eating vegetables			**0.000**
Twice a day or more often	1,471 (40)	1,513 (44)	
Once a day	1,364 (37)	1,305 (38)	
Less than once a day	843 (23)	606 (18)	
Eating fruit/berries			0.393
Twice a day or more often	1,839 (50)	1,727 (50)	
Once a day	1,433 (39)	1,352 (39)	
Less than once a day	413 (11)	350 (10)	
Eating fish			**0.023**
Twice a week or more often	1,330 (36)	1,339 (39)	
Once a week	1,721 (47)	1,559 (46)	
Less than once a week	610 (17)	513 (15)	
Eating sweets/drinking soft drinks			0.353
Once a week or less often	1,616 (44)	1,447 (42)	
A few times a week	1,924 (53)	1,848 (54)	
Once a day or more often	125 (3)	115 (3)	
Tooth brushing			**0.000**
Twice a day or more often	2,964 (80)	2,869 (84)	
Once a day or less often	718 (20)	546 (16)	
Playing outdoors, weekdays			**0.001**
≥3 h	1,800 (51)	1,547 (47)	
<3 h	1699 (49)	1,717 (53)	
Playing outdoors, weekends			**0.000**
≥3 h	1,904 (54)	1,573 (48)	
<3 h	1,629 (46)	1,706 (52)	
Sedentary screen time, weekdays			0.170
≤ 1 h	2,308 (65)	2,194 (66)	
>1 h	1,254 (35)	1,111 (34)	
Sedentary screen time, weekends			0.299
≤ 1 h	1,095 (31)	1,057 (32)	
>1 h	2,487 (69)	2,274 (68)	
Reading to the child			0.066
Every day	2,354 (64)	2,268 (66)	
A few times a week	1,051 (28)	937 (27)	
Once a week or less often	297 (8)	237 (7)	

a *Chi-squared test was used to report significant differences between boys and girls. P < 0.05 was considered as significant and marked with bold values*.

### Statistical Analyses

This study used cross-sectional descriptive and comparative statistical analyses. The distribution of responses was calculated for girls and boys, respectively. Pearson's chi-squared test was used to analyze gender differences for all categorical variables. To analyze the overall associations between social-emotional problems and the explanatory variables among 3-year-olds, simple and multiple logistic regression analyses were performed, and odd ratios (OR) and Confidence Intervals (CI) were reported. A backward elimination procedure was performed until only significant variables (*p* < 0.05) were left in the final analyses. The data was analyzed using Stata/SE version 16.0 (StataCorp, College Station, Texas 77,845 US).

### Ethical Considerations

Only children whose parents have given written informed consent were included in the study. The Regional Ethical Review Board in Umeå approved the study (2013-268-31O).

## Results

A majority of the questionnaires were completed by the parents jointly (62%) and the remaining by mothers alone (34%), fathers alone (4%) or by another person (1%). Characteristics of the 3-year-olds and gender differences are shown in Table 1. Most of the 3-year-olds had parents living together (boys = 91%, girls = 92%), three out of four had siblings and almost all 3-year-olds attended preschool (mean 29 h per week, SD 11). When it comes to lifestyle, a high proportion of the 3-year-olds had markers of unhealthy lifestyle, more so for boys than for girls and gender differences were evident. Parents reported that boys less often drank low-fat milk, ate vegetables and fish and less frequently had parent/-s who brushed their teeth and read to them compared to girls (*p* < 0.05). On the other hand, boys spent more hours playing outdoors, both during weekdays and weekends compared to girls (*p* < 0.001).

There were statistically significant associations between most of the lifestyle characteristics and children's social-emotional problems (*p* < 0.05) as shown in Table 2. However, some gender differences appeared here as well. Not having siblings, drinking low-fat milk less than once a day, <3 h playing outdoors during the weekdays and ditto during the weekends increased the risk of social-emotional problems only for boys, while eating fruit/berries once a day or less than once a day were significant only for girls.

**Table 2:** Associations between 3-year-olds' lifestyle and social-emotional problems as measured by ASQ:SE^a^ using simple logistic regressions.

**Characteristics**	**Boys OR (95% CI)**	***P*-value^**b**^**	**Girls OR (95% CI)**	***P*-value^**b**^**
Custody arrangement				
Parents living together	1		1	
Parents not living togetheror single parent	1.8 (1.3-2.4)	**0.000**	2.5 (1.7-3.8)	**0.000**
Having siblings				
Yes	1		1	
No	1.4 (1.1–1.7)	**0.004**	1.1 (0.8–1.5)	0.718
Attending preschool				
Yes	1		1	
No	1.0 (0.5–1.7)	0.936	0.5 (0.1–1.9)	0.284
Drinking low-fat milk				
Twice a day or more often	1		1	
Once a day	1.1 (0.8–1.4)	0.568	1.3 (0.9–2.0)	0.129
Less than once a day	1.6 (1.3–2.1)	**0.000**	1.4 (1.0–2.1)	0.082
Eating vegetables				
Twice a day or more often	1		1	
Once a day	1.3 (1.0–1.7)	**0.017**	1.2 (0.9–1.8)	0.220
Less than once a day	1.9 (1.5–2.5)	**0.000**	2.5 (1.8–3.6)	**0.000**
Eating fruit/berries				
Twice a day or more often	1		1	
Once a day	0.9 (0.7–1.1)	0.260	1.4 (1.0–1.9)	**0.037**
Less than once a day	1.2 (0.8–1.6)	0.311	2.0 (1.3–3.2)	**0.001**
Eating fish				
Twice a week or more often	1		1	
Once a week	1.2 (1.0–1.6)	0.059	1.1 (0.8–1.6)	0.405
Less than once a week	1.5 (1.1–2.0)	**0.005**	2.4 (1.7–3.6)	**0.000**
Eating sweets/drinking soft drinks				
Once a week or less often	1		1	
A few times a week	1.1 (0.9–1.3)	0.418	1.0 (0.7–1.3)	0.981
Once a day or more often	1.9 (1.2–3.0)	**0.007**	2.1 (1.1–3.9)	**0.026**
Tooth brushing				
Twice a day or more often	1		1	
Once a day or less often	2.3 (1.8–2.8)	**0.000**	2.3 (1.7–3.2)	**0.000**
Playing outdoors, weekdays				
≥3 h	1		1	
<3 h	1.4 (1.1–1.7)	**0.003**	1.3 (1.0–1.8)	0.103
Playing outdoors, weekends				
≥3 h	1		1	
<3 h	1.3 (1.1–1.6)	**0.015**	1.2 (0.9–1.7)	0.207
Sedentary screen time, weekdays				
≤ 1 h	1		1	
>1 h	1.5 (1.2–1.8)	**0.000**	2.2 (1.6–2.9)	**0.000**
Sedentary screen time, weekends				
≤ 1 h	1		1	
>1 h	1.5 (1.2–1.9)	**0.000**	1.6 (1.2–2.3)	**0.006**
Reading to the child				
Every day	1		1	
A few times a week	1.4 (1.1-1.7)	**0.005**	1.6 (1.1-2.2)	**0.004**
Once a week or less often	1.9 (1.4-2.6)	**0.000**	3.4 (2.2-5.2)	**0.000**

a *The first edition of the Ages and Stages Questionnaires: Social-Emotional (ASQ:SE) for 36-month interval*.

b *P < 0.05 was considered as significant and marked with bold values. Logistic regression was used to assess the association between each explanatory variable and 3-year-olds' social-emotional problems*.

**Figure 1:**
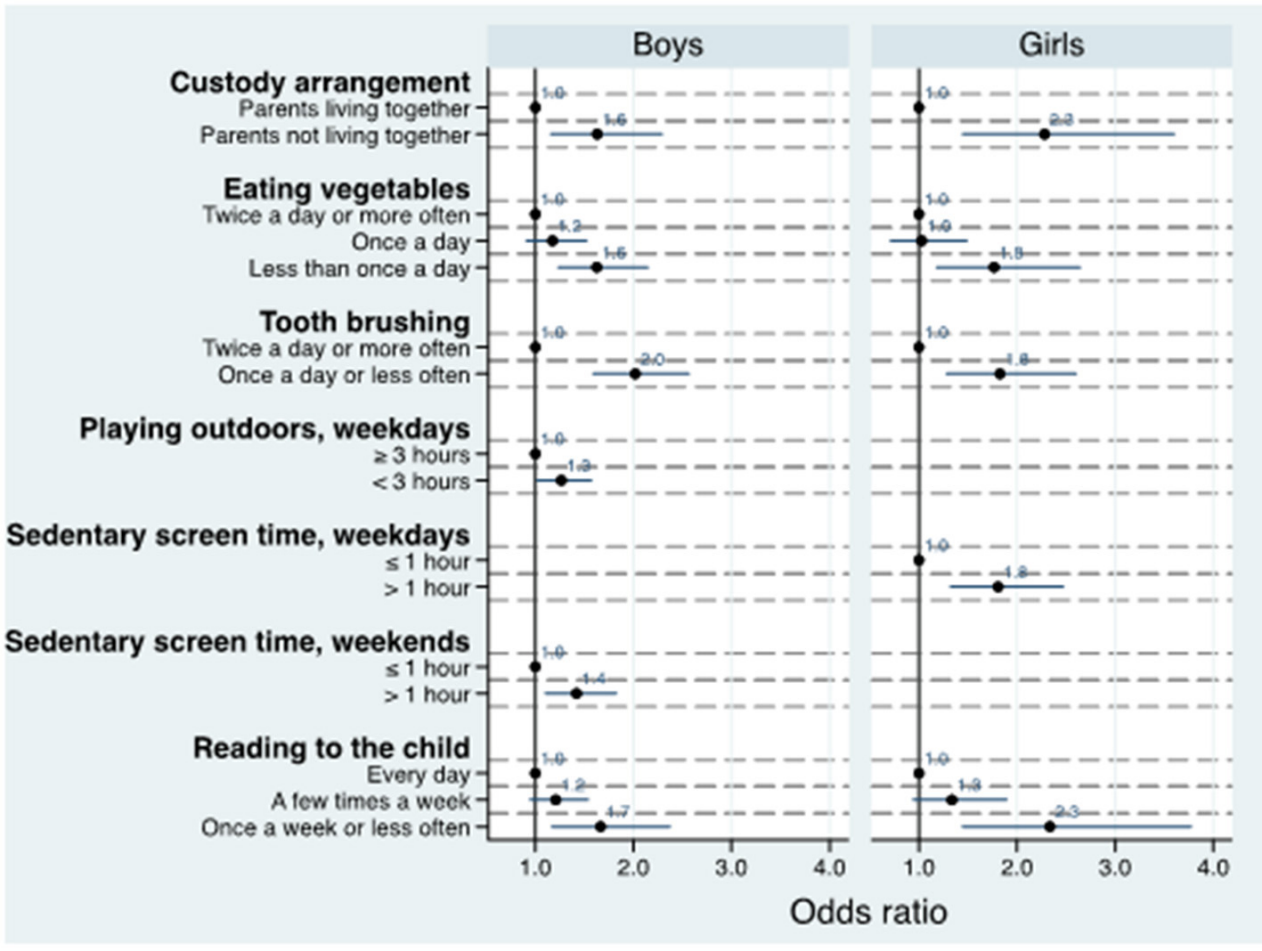
Adjusted estimates^a^ of the association between 3-year-olds' lifestyle and social-emotional problems as measured by ASQ:SE^b^. ^a^Multiple logistic regression with backward elimination included only variables that were still significant in the final analyses. ^b^The first edition of the Ages and Stages Questionnaires: Social-Emotional (ASQ:SE) for 36-month interval.

After multiple logistic regression with backward elimination (Figure 1), higher risk of social-emotional problems were found among children without parents living together, with less frequently vegetable consumption and among those children whose parent/-s brushed their teeth and read to them less often. However, less time for playing outdoors and more sedentary screen time during weekends increased the risk of social-emotional problems among boys only, while more sedentary screen time during weekdays increased the risk of social-emotional problems among girls only.

## Discussion

In this study, we found significant associations between most of the lifestyle characteristics and children's social-emotional problems, which gives us an overall picture of the family life situation of children with such problems. A high proportion of the 3-year-olds had an unhealthy lifestyle, more so for boys than for girls. In general, an unhealthy lifestyle increased the children's risk of having social-emotional problems measured by ASQ:SE and using the recommended cut-off (1). Below we discuss our findings, one lifestyle characteristic at a time, and implications for practice and research are proposed.

Our findings suggested that *custody arrangement* was associated with social-emotional problems among the children. Children who did not live with both parents were more likely to be at risk, especially girls. We have discussed this finding in detail in our previous publication (7). The same applies to the *gender differences* we found. Parents of boys more often reported social-emotional problems than parents to girls (7), which is similar to other previous studies of gender differences in preschool-aged children (5, 25, 26).

Overall, lifestyle of the child might mirror the family's life situation, as there were significant associations between many unhealthy lifestyle characteristics and children's social-emotional problems. We do not claim any causal relationship between lifestyle and children's social-emotional problems, as we with this cross-sectional study design cannot clarify which comes first, the unhealthy lifestyle or the social-emotional problems. However, our findings suggest that children are more likely to have social-emotional problems in a context in which unhealthy lifestyle is prevailing.

The dietary quality in terms of *food-frequency* reported in our study were somewhat more adequate for the girls than for the boys regarding drinking low-fat milk and eating fish and vegetables as reported by their parents. Almost 70% of the children drank the recommended frequency of low-fat milk, but only half when it came to eating fruit/berries. Even fewer were eating the recommended frequency of vegetables and fish. The gender differences in dietary quality in our study are in accordance with studies during pregnancy, where women generally have healthier food habits than men (27). In schoolchildren, gender differences seems to be rare, but girls seems to like fruit and vegetables more compared to boys (28).

In girls, it was more likely to have social-emotional problems among those who ate insufficient frequencies of vegetables, fish and fruit/berries. For vegetables, the results remained significant in the multiple logistic regression analysis for both boys and girls. This is in accordance with two different systematic reviews that concluded that healthy foods such as vegetables, salads, fruit and fish, are associated with better mental health of children and adolescents, and to some extent the reverse was found (29). Increased intake of high-sugar products and lower diet quality have been reported to be associated with higher likelihood of emotional symptom in children (30, 31). Interestingly, no study had included children below 4.5 years, highlighting that this study contributes with new knowledge about 3-year-olds' dietary quality and the association with social-emotional problems.

When it comes to fulfillment of the recommended *tooth brushing* twice a day, we found the same gender pattern among adults during pregnancy (27) and adolescents (32) as among the 3-year-olds in our study, where fewer boys than girls fulfilled the recommendation. Thus, it seems as parents transfers their tooth brushing habits to their child in a gendered mode, thus, treating girls and boys differently. Another explanation could be that boys have more social-emotional problems, and additionally, their expressions of such problems are more externalized than those of girls (23), and therefore more difficult to manage for the parents. Besides that, there were considerably higher risk of social-emotional problems among both boys and girls whose teeth were brushed less than twice a day. Another study with parents from poor areas have reported that the main barriers for brushing their children's teeth regularly were skills in managing their children's behavior and their stressful lives (33). Eating patterns and tooth brushing are important for dental health and have additive impact on prevention of caries (34). In Sweden, dental hygienists are meeting all children regularly, often accompanied by a parent. This is particularly important for promoting equal dental and oral health among children, although there are measurable differences between boys and girls in frequency of tooth brushing as shown in our study.

The variable *playing outdoors* used in our study was chosen as a proxy for the recommended physical activity at any intensity for 180 min, of which at least 60 min should be moderate to vigorous, spread throughout the day (35). More than half of the boys and less than half of the girls were playing outdoors during weekdays as well as weekends. These gender differences in physical activity found in our study are in accordance with previous studies at preschool age (36–38) and the same pattern is seen in adolescence. Playing outdoors for <3 h during weekdays increased the risk of social-emotional problems among boys but not girls in our study. A study of children aged 1 to 5 years found that increased temperamental behaviors were associated with less time playing outdoors among boys, but not among girls (39).

WHO has clarified that *sedentary screen time* is spent passively watching screen-based entertainment (TV, computer, mobile devices) which does not include active screen-based games for which physical activity or movement is required (35). We have considered this aspect in our study while asking for sitting in front of the screen. The recommended maximum limit from WHO for sedentary screen time is 1 h per day for those aged 3–4 years (35), and two-thirds of both boys and girls fulfilled that recommendation. More than 1-h of sedentary screen time during weekends increased the risk of social-emotional problems among boys, and the same was true during weekdays among girls in our study. The time spent in outdoor play and sedentary screen time was a little <3 and 2 h, respectively (data not shown) in accordance with a recent study of proxy-reported data (36). Another study showed further interesting findings in line with our results, that preschool children's social skills are adversely associated with screen time and favorably associated with outdoor play (36).

In the adjusted analyses, both girls and boys whose *parent/-s read to them* seldom were about two times more likely to have social-emotional problems. A randomized controlled trial of children up to the age of three found that reading has important positive effects on social-emotional development (40) and the authors recommended this as a primary prevention of social-emotional problems. Our study result could be looked through two lenses; that children develop social-emotional problems because their parent/-s do not give them enough attention, including lack of reading, *or* parents of children with social-emotional problems do not read to their children because their overall family life situation is difficult to handle, including the problems of the child. The latter is supported by a study which demonstrates a close link between mothers' psychological distress and their ASQ:SE ratings of infant's (41). This means that on the one hand, high total scores can reflect the parent's mental health problems more than the child's ditto can, on the other hand, a healthy child can be negatively affected if the parent has such problems.

The current study is population-based with high participation rate, as almost hundred percent of parents and their child attend the 3-year's health check-up at the Child Health Care center. We used the well-established ASQ:SE, which has been shown to be an appropriate tool to investigate children's social and emotional problems (21, 22). Since ASQ:SE is so far only validated in an U.S. context (1) it brings a limitation into our study. The cut-off used (59 points) may not be optimal in the Swedish context, although it is used in many other countries. In a recent Nordic review of the evidence of reliability, validity and norms of different psychological tests including ASQ:SE, the reliability was considered satisfactory, although there is a lack of Nordic up-to-date norming and validation studies (42).

The present study used both the English and the Swedish versions of the ASQ:SE, which means that non-Swedish speakers who have English as their first or second language could still answer the questionnaire. However, parents who do not have strong skills in any of these languages were not able to answer the questionnaire, which can be assumed to have contributed to a lower proportion of immigrants in our study. In addition, the data collection was done through parent/-s' assessment of their child's behavior. On one hand, this is a strength, as most parents know their child best, on the other hand, as already mentioned, parent's own problems and wishes may also reflect how they report their child's abilities and problems, as parents' well-being directly affects that of their children (43, 44).

There are both strengths and weaknesses with proxy reports and markers of lifestyle characteristics compared to objectively measured data. The reports do not go into details, but gives us an overview of the child's lifestyle. On the other hand, we don't know the daily amount of food intake or the exact frequency, duration and intensity for physical activity. Most 3-year-olds were in preschool with a mean duration of 29 h. At preschool breakfast, snacks (at morning and afternoon) and lunch are served and contains fruit, berries, vegetables and low-fat milk daily, while fish is served about once a week. They also spend time playing outdoors before and/or after lunch. Knowledge of food-frequency and time playing outdoor may be difficult for parents to report exactly. When it comes to physical activity and playing outdoors, we do not know the type of the activity either. From other research on children living in Sweden, we know that children spend more time playing indoors than outdoors (38), and we only measured outdoor activity in this study. However, it is rather difficult to do any more than light physical activity indoors, both at home and in preschool on a limited space, and the health benefits of light physical activity is unknown. In the light of studies with objective measurement methods, our proxy reports appear to show an overestimated time in outdoor play and an underestimated screen time (37, 38). Sedentary screen time, which is also difficult to report, is an ambiguous variable that can include the positive aspects of engaging in reading and storytelling on a screen with a caregiver (35). Finally, custody arrangement has been used as a reflection of socioeconomics, since no other ones were included in the analyses. It would be helpful in further studies to bring in data on ethnicity and socioeconomic status, as it has been recommended in research on child health (45).

In order to support children with social-emotional problems, our study calls for intervention programs with a broader perspective on improving children's lifestyle rather than merely focusing on their social and emotional health. Today, there is a major focus on improving mental health and lifestyle in public health, but often as two separate tracks. This study demonstrates the importance of considering both as these are intertwined. Gender differences in lifestyle and in the development of social-emotional health deserve attention and should be highlighted within Child Health Care. As nurses are the main professional meeting the parents' and their child through the first critical 5 years in Swedish Child health services, they are key for promoting physical activity, healthy food habits and preventing sedentary behavior for being established in early childhood (46). This also applies to other lifestyle characteristics and social-emotional development. To strengthen and promote a healthy lifestyle in preschool-age children is a challenge as multiple factors seem to influence physical activity and sedentary behaviors at various levels, including intrapersonal, interpersonal, environmental, organizational, and policy (46).

## Conclusions

Our study provides us with an important overview picture of the family life situation of 3-year-olds, including those with social-emotional problems. Such problems were significantly associated with markers of unhealthy lifestyle, with significant gender differences, already at 3-years-of age. Therefore, this study suggests that in order to maintain children's social-emotional ability and support children at risk of problems, public health intervention programs should have a broader perspective on improving children's lifestyle rather than merely focusing on their social and emotional problems. The gender differences found may be taken in account.

## Data Availability Statement

The raw data supporting the conclusions of this article will be made available by the authors after application to Umeå University and approval by the Regional Ethical Review Board. Requests to access the datasets should be directed to eva.eurenius@umu.se.

## Ethics Statement

The studies involving human participants were reviewed and approved by the Regional Ethical Review Board, Umeå University, SE-901 87 Umeå, Sweden. Written informed consent to participate in this study was provided by the participants' legal guardian/next of kin.

## Author Contributions

EE, AM, ML, AI, and MV designed the study. EE was responsible for data collection and designed the questions in collaboration with AI and finally revised the manuscript after all authors had critically reviewed the manuscript. MV, ML, and EE prepared the data for analysis. AM analyzed and interpreted the data in collaboration with ML and MV. EE, AM, and ML drafted the initial manuscript. AI and IÖ contributed in interpretation and editing of the manuscript. All authors have read and approved the final manuscript for submission.

## Funding

Both Region Västerbotten and Umeå University contributed in making this study possible.

## Conflict of Interest

The authors declare that the research was conducted in the absence of any commercial or financial relationships that could be construed as a potential conflict of interest.

## Publisher's Note

All claims expressed in this article are solely those of the authors and do not necessarily represent those of their affiliated organizations, or those of the publisher, the editors and the reviewers. Any product that may be evaluated in this article, or claim that may be made by its manufacturer, is not guaranteed or endorsed by the publisher.
